# A new family of periplasmic-binding proteins that sense arsenic oxyanions

**DOI:** 10.1038/s41598-018-24591-w

**Published:** 2018-04-19

**Authors:** Consuelo Badilla, Thomas H. Osborne, Ambrose Cole, Cameron Watson, Snezana Djordjevic, Joanne M. Santini

**Affiliations:** 1 Institute of Structural & Molecular Biology, Division of Biosciences, University College London, London, WC1E 6BT UK; 2 Institute of Structural & Molecular Biology, Department of Biological Sciences, Birkbeck College, University of London, WC1E 7HX London, UK

## Abstract

Arsenic contamination of drinking water affects more than 140 million people worldwide. While toxic to humans, inorganic forms of arsenic (arsenite and arsenate), can be used as energy sources for microbial respiration. AioX and its orthologues (ArxX and ArrX) represent the first members of a new sub-family of periplasmic-binding proteins that serve as the first component of a signal transduction system, that’s role is to positively regulate expression of arsenic metabolism enzymes. As determined by X-ray crystallography for AioX, arsenite binding only requires subtle conformational changes in protein structure, providing insights into protein-ligand interactions. The binding pocket of all orthologues is conserved but this alone is not sufficient for oxyanion selectivity, with proteins selectively binding either arsenite or arsenate. Phylogenetic evidence, clearly demonstrates that the regulatory proteins evolved together early in prokaryotic evolution and had a separate origin from the metabolic enzymes whose expression they regulate.

## Introduction

**Figure 1:**
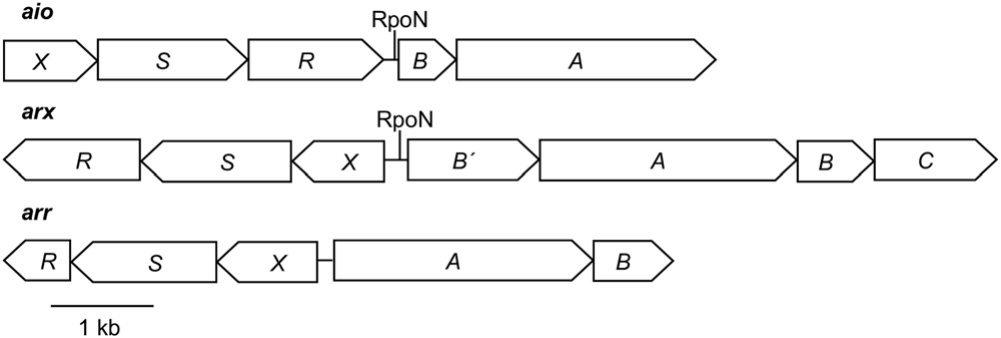
Gene arrangements for the arsenite oxidase (*aio*), alternative arsenite oxidase (*arx*) and respiratory arsenate reductase (*arr*) gene clusters. The *aio* represented is from *Rhizobium* sp. str. NT-26, *arx* from *Alkalilimnicola ehrlichii* str. MLHE-1 and *arr* from *Chrysiogenes arsenatis*. *A*: molybdenum-containing subunit; *B*: iron-sulphur protein; *C*: integral membrane subunit; *R*: response regulator; *S*: sensor histidine kinase; *X*: oxyanion-binding subunit. Putative promoter sites for RpoN are indicated, we could not detect a promoter upstream of *arrAB*.

Arsenic, although toxic to most living organisms, can be used for respiration by some prokaryotes. Under anoxic conditions, arsenate (+V) can serve as a terminal electron acceptor coupling its reduction to the oxidation of a variety of organic and inorganic electron donors^1^. With oxygen or nitrate (at pH > 9) as the terminal electron acceptor, arsenite (+III) can be used as an electron donor with some organisms clearly gaining energy from its oxidation^2,3^. In all cases of arsenic metabolism, the metabolic enzymes involved in the oxidation or reduction reactions are members of the DMSO reductase family of molybdenum-containing enzymes^4–8^. The most well characterised arsenic-metabolising enzyme is the arsenite oxidase (Aio) purified from aerobic arsenite oxidisers, with two X-ray crystal structures resolved^9,10^. Aio consists of two heterologous subunits, a catalytic molybdopterin guanine dinucleotide-containing subunit (AioA) that also contains a 3Fe-4S cluster, and a small Rieske subunit (AioB)^9^. The respiratory arsenate reductase (Arr) has been purified and characterised from three organisms^6,8,11^ and like the Aio consists of two heterologous subunits, a molybdenum-containing subunit (ArrA), which based on sequence analysis contains one 4Fe-4S cluster and a small subunit (ArrB) with four 4Fe-4S clusters^8^. The alternative arsenite oxidase, Arx, which operates in an anaerobic respiratory chain with nitrate reductase as the terminal reductase, has not been studied biochemically but is thought to consist of four subunits based on the *arx* loci (Fig. 1). Two of the gene products (ArxA and ArxB) have a high degree of sequence similarity to ArrA/B suggesting an identical cofactor composition; in Arx there is also another putative subunit that contains six predicted 4Fe-4S clusters (ArxB′) and an integral membrane protein (ArxC)^5,12,13^.

In some arsenic-metabolising bacteria, expression of the molybdoenzyme is strictly regulated, with gene expression only detected when the organism is grown in the presence of the arsenic oxyanion^2,6,12,14–16^. The expression of Aio in the *Proteobacteria* is regulated by a regulatory gene cluster adjacent to the arsenite oxidase genes (*aioBA*). The regulatory cluster consists of three genes, *aioX*, *aioS* and *aioR*, which encode a periplasmic-binding protein (PBP), a sensor histidine kinase and a response regulator, respectively. All three genes are essential for arsenite oxidase gene (*aioBA*) expression^14,16–19^ and in the case of *Rhizobium* sp. str. NT-26 are co-transcribed^20^. AioX is thought to be the sensing protein as it has been shown to bind arsenite *in vitro*^14^. Arsenite-bound AioX is thought to act as a ligand for AioS, the sensor histidine kinase that upon auto-phosphorylation, phosphorylates AioR, the response regulator, which binds to a conserved sequence upstream of a RpoN promoter^14,15,18,19^.

Here we report the identification and characterisation of a new subfamily of arsenic oxyanion-binding proteins from phylogenetically distant bacteria that have evolved to regulate bioenergetic arsenic metabolism. These proteins, which demonstrate substrate selectivity, could be used as novel sensors for both toxic arsenic oxyanions.

## Results

### Conservation of the *XSR* regulatory gene cluster

The *aioXSR* regulatory gene cluster is found in some arsenite oxidisers of the *Alpha-* and *Betaproteobacteria*. Searches of the GenBank database, revealed that the *XSR* regulatory module was conserved in a sub-set of other phylogenetically distant arsenic-metabolising bacteria including the anaerobic arsenite oxidiser, *Alkalilimnicola ehrlichii* str. MLHE-1 (*Gammaproteobacteria*) (encode Arx – genes are *arxB*′*ABC*) and the arsenate respirer, *Chrysiogenes arsenatis* (*Chrysiogenetes*) (encode Arr – genes are *arrAB*) (Fig. 1). This finding suggests that the XSR proteins in these organisms regulate expression of the respective molybdenum-containing enzyme.

AioR and ArxR are the response regulators controlling expression of the arsenite oxidase genes and are members of the AtoC family of response-regulators (similar structure to the response regulator for acetoacetate sensing in *Escherichia coli*^21^), which contain a AAA+ ATPase domain. Like in NT-26, a putative RpoN promoter (tggcaaggctattgct) and ArxR-binding site (gtaacaattcaagc) (based on homology to the NT-26 AioR-binding site), were identified upstream of *the* arsenite oxidase genes. ArrR, is a member of a different response regulator family, namely the CitB-like family, similar to the response regulator for citrate sensing in *Klebsiella pneumoniae*^22^ and unlike AioR/ArxR does not possess an AAA+ ATPase domain. ArrR has sequence similarity to the DNA-binding, helix-turn-helix portion of FixJ^23^, with a putative FixJ-like binding site motif^24^ (tcaggtttttccctga) identified upstream of *arrA*.

### Regulatory modules and molybdenum-containing subunits have different phylogenies

**Figure 2:**
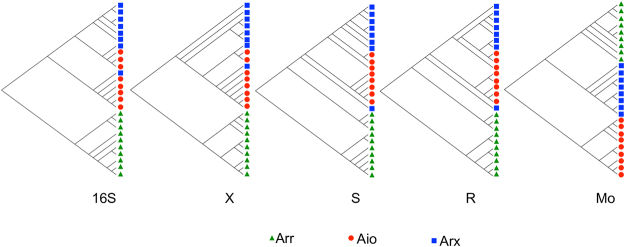
Phylogeny of arsenic-metabolising bacteria, metabolic enzymes and regulatory proteins. Trees are for 16S rRNA gene (16S); AioX homologues (X); AioS homologues (S); AioR homologues (R); and the molybdenum-containing subunits of Aio, Arx and Arr (Mo). Trees are bootstrap consensus trees constructed using the maximum-likelihood method from 200 resamples and are rooted using the 16S rRNA gene from *Sulfolobus tokodaii*, and the protein sequences from TorT, TorS, TorR and TorA for X, S, R and Mo, respectively.

The phylogeny of the XSR regulatory modules are congruent with the 16S rRNA gene phylogeny (Fig. 2; annotated 16S rRNA tree as Fig. S2). By contrast, in the tree for the molybdenum-containing subunit of the metabolic enzymes, Aio forms a separate clade from Arx and Arr (Fig. 2). The trend is exemplified by the Arx regulatory proteins from the Alphaproteobacterium *Magnetospirillum magnetotacticum*, where the 16S rRNA gene, ArxX, ArxS and ArxR sequences all cluster with other *Alphaproteobacteria*, which are all aerobic arsenite oxidisers (Fig. 2). The ArrR from the *Epsilonproteobacteria* are more similar (i.e. they are in the AtoC family) to ArxR and AioR than to the ArrR from the *Firmicutes* and *Chysiogenetes*, which is a further example of how the XSR phylogeny is more congruent to the 16S rRNA gene than to that of the molybdenum-containing enzyme.

The genes that encode the alternative arsenite oxidase (*arxB’ABC*) together with the regulatory cluster (*arxXSR*) are conserved in anaerobic arsenite oxidisers, all of which are members of the *Alpha-*, *Beta-* and *Gammaproteobacteria*. The *aioBA* genes are more widespread, with homologues in both prokaryotic domains of life and in the following phyla of the Bacteria, *Proteobacteria, Chloroflexi* and *Thermus-Deinococcus*. The *aioXSR* regulatory module, however, is present only in some arsenite oxidisers in the *Alpha-* and *Betaproteobacteria*. In the arsenate respirers, the *arrAB* genes are present in members of the *Firmicutes, Proteobacteria (Gamma- and Epsilonproteobacteria), Chrysiogenetes* and *Deferribacteres* phyla while the *arrXSR* genes are present in all of these except the *Gammaproteobacteria* and the *Deferribacteres*.

### Phylogeny and evolutionary history of AioX

**Figure 3:**
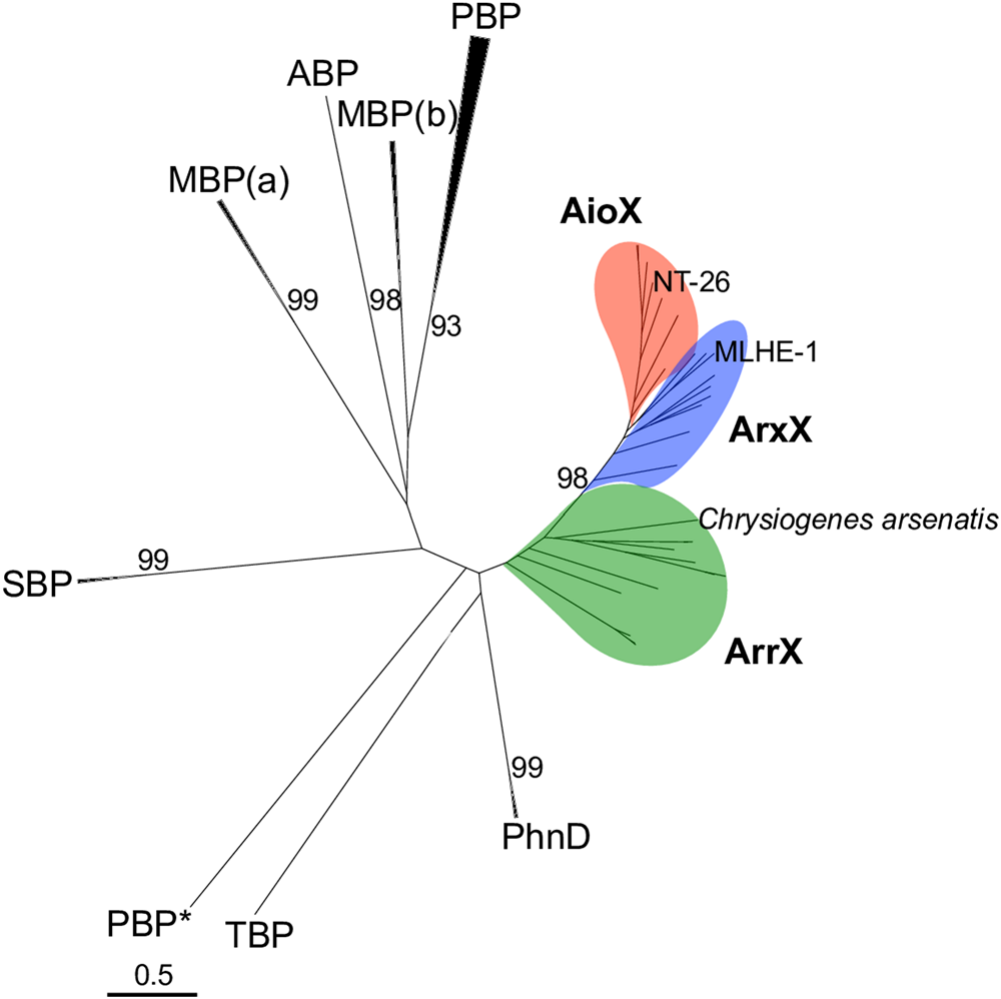
Maximum-likelihood phylogenetic tree of AioX and its homologues. ABP, antigen-binding protein; AioX (red) and ArxX (blue), arsenite-binding proteins; ArrX, (green), arsenate-binding proteins; MBP, molybdate-binding proteins (a: archaeal; b: bacterial); PBP, phosphate-binding proteins (*involved in sensing not transport); PhnD, phosphonate-binding proteins; SBP: sulphate-binding proteins; TBP: tungstate-binding protein.

Based on sequence comparisons, AioX appears to represent a new subfamily of the PBPs^25^ where the majority of the proteins bind tetrahedral oxyanions, including phosphate, sulphate, molybdate and tungstate^26^ (Fig. 3). The new subfamily is distinct from the other proteins as it functions in sensing and not oxyanion transport^26–28^. As expected, the closest homologues include ArxX and ArrX presumed to be involved in sensing arsenite and arsenate, respectively.

### Three-dimensional structure of AioX

**Figure 4:**
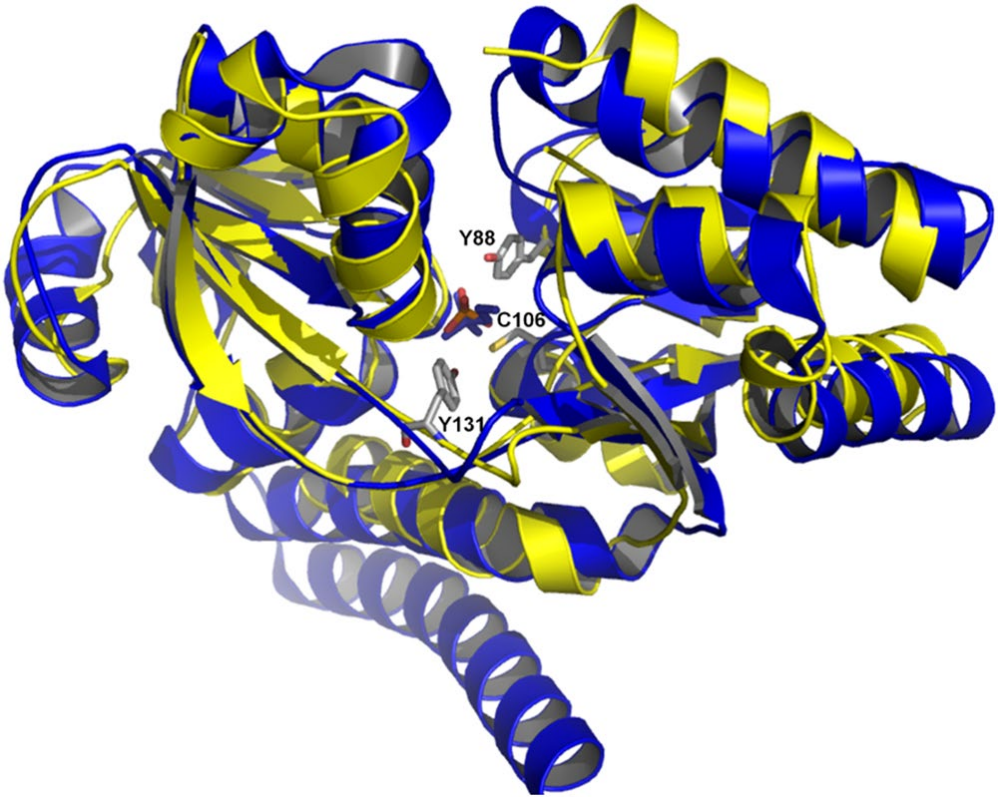
Structure of AioX superimposed with that of PhnD. The bilobal structure of AioX is shown in ribbon representation (yellow) and compared to that of the *E. coli* protein PhnD in complex with its ligand 2-aminoethyl phosphonate (blue ribbons and blue sticks, respectively). The AioX structure shown is that of the SAD method solved structure, which contains phosphate in its binding pocket. Two conserved tyrosine residues (Y88 and Y131), AioX-specific residue C106, and a phosphate ion are shown as sticks and coloured based on the type of the atom (phosphorous-orange, oxygen-red, nitrogen-blue, and carbon-grey).

Structures of the apo, arsenite-bound and phosphate-bound forms of AioX were solved and deposited in PDB with the following IDs: 6ESK, 6EU7 and 6ESV, respectively. X-ray data and refinement statistics are presented in Table 1. AioX exhibits a typical bilobed structure of the PBP type II class^25^. The 304 amino acid residue polypeptide folds into two similar α/β domains. Each domain contains mixed beta-sheets, one with 6 strands and the other with 5; one strand is antiparallel to the rest in each domain (Fig. 4). The two domains are linked by two crossing-over protein strands, such that one links the beta strand 4 of the first domain to the beta strand 1 of the second domain, while the second crossover links the beta strand 5 of the second domain back to the beta strand 5 of the first domain. In addition, there is a C-terminal extension to the canonical fold, which is the sixth beta strand in the first domain and a C-terminal helix.

**Table 1:** Data collection and refinement statistics.

Protein (AioX)	Apo	Arsenite-bound	Phosphate-bound
PDB ID	6ESK	6EU7	6ESV
**Data collection**			
Space Group	P 43 21 2	C 2	P 43 21 2
**Cell dimensions**			
a, b, c (Å)	124.88, 124.88, 47.73	202.36, 116.92, 136.84	124.5, 124.5, 48.01
α, β, γ (°)	90.0, 90.0, 90.0	90.0, 90.02, 90.0	90.0, 90.0, 90.0
Resolution (Å)	44.59–1.75 (1.78–1.75)^**a**^	44.44–3.00 (3.07–3.00)	44.03–1.78 (1.82–1.78)
R_merge_	0.380 (12.334)^**b**^	0.11 (0.587)	0.109 (2.036)
Completeness (%)	98.9 (81.0)	97.9 (99.0)	99.8 (100)
Multiplicity	12.8 (9.1)	4.0 (4.1)	16.4 (16.6)
CC_1/2_	0.996 (0.126)^**b**^	0.993 (0.651)	0.998 (0.740)
Mean((I)/sd(I))	7.6 (0.4)^**b**^	10.0 (2.5)	19.3 (2.1)
**Refinement**			
Total number of observations	492945	249664	603454
Total number unique	38474	62521	36718
R_work_/_free_	0.19/0.21	0.19/0.21	0.19/0.22
**RMS deviations**			
Bonds (Å)	0.022	0.014	0.021
Angles (°)	1.969	1.659	2.008
Mean B-factors (Å^2^)	29.7	61.3	31.7
**Ramachandran plot (%)**			
Favoured	98.1	95.4	97.3
Allowed	1.5	4.4	2.7
Outliers	0.4	0.3	0

^a^Data for the highest resolution shell is shown in parenthesis. ^b^Effective resolution of this data set based on CC_1/2_ and Mean((I)/sd(I)) is1.85 Å and 1.97 Å, respectively. R_merege_ at 1.97 Å is 0.267. All data was used in the refinement.

The AioX topology closely resembles that of the *E. coli* phosphonate-binding protein PhnD (Fig. 4). The NT-26 AioX and the *E. coli* PhnD proteins share 20% sequence identity over 245 aligned residues while the two structures superpose with a core rmsd of 1.84 Å, indicating close structural homology. In this class of proteins, the ligand-binding pocket is located in the space between the two domains.

In the initial single-wavelength anomalous diffraction (SAD) electron density maps generated from the crystals of Se-Met labelled protein there was an unaccounted electron density in the putative ligand-binding region near residue Cys106. The electron density was consistent with the presence of a phosphate ion, from the crystallisation conditions, and its location would suggest that Cys106 might be involved in binding arsenite.

### Arsenite-bound AioX displays significant conformational changes

**Figure 5:**
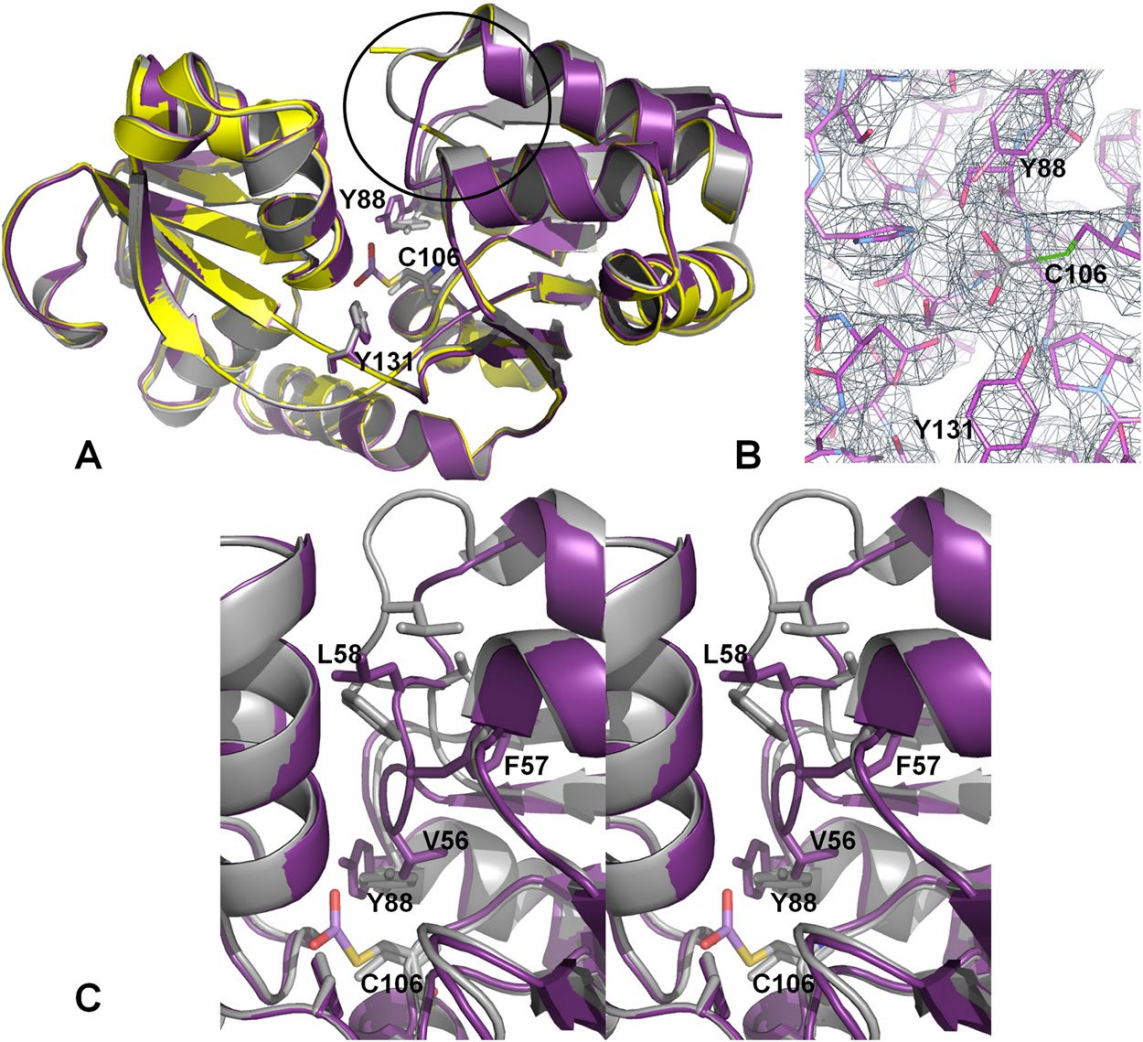
Conformational changes associated with arsenite-binding. The structures of the apo (grey), arsenite-bound (purple) and phosphate-bound (yellow) forms of AioX were overlaid. (**A**) Arsenite-binding causes the Y88 side chain rotamer to change in concert with a large backbone conformational change spanning residues 53–61 (circled in the Figure). The electron density for the corresponding region in the phosphate-bound AioX crystal structure was disordered. (**B**) Electron density map showing the linkage between the bound arsenite and Cys106. (**C**) Stereo diagram showing a closer view of the loop conformational change with the three hydrophobic residues closing in on the ligand-binding site. The figure was created without the smoothing function for the backbone representation emphasizing the full extent of the backbone conformational changes associated with arsenite binding.

The crystal structure of the phosphate-bound AioX was used to solve the structures of the apo and arsenite-bound forms of the protein by molecular replacement. Figure 5 shows the AioX ligand-binding site with arsenite forming a link to the sulphur atom of Cys106, confirming the role of this residue in arsenite binding. Figure 5 also shows an overlay of the apo and the arsenite-bound forms of AioX accentuating the most pronounced differences between the two structures. While the majority of both structures overlay very well, there is a significant difference in the rotamer conformation of Tyr88. The change in the side chain conformation is further associated with the backbone shift for residues 83–88 and a significant conformational change in the loop region comprising residues 53–61, demarked by the black circle in Fig. 5A. This conformational change upon arsenite binding involves a hydrophobic collapse of the loop, with residues Val56, Phe57 and Leu58 reaching towards the core of the protein and closing of the ligand-binding site. Importantly, the conformation of Tyr88, seen in the apo form of AioX and the conformation of the 53–61 loop, observed in the arsenite-bound AioX, are mutually exclusive, as the conformations of these two parts of the structure would have caused a steric clash.

**Figure 6:**
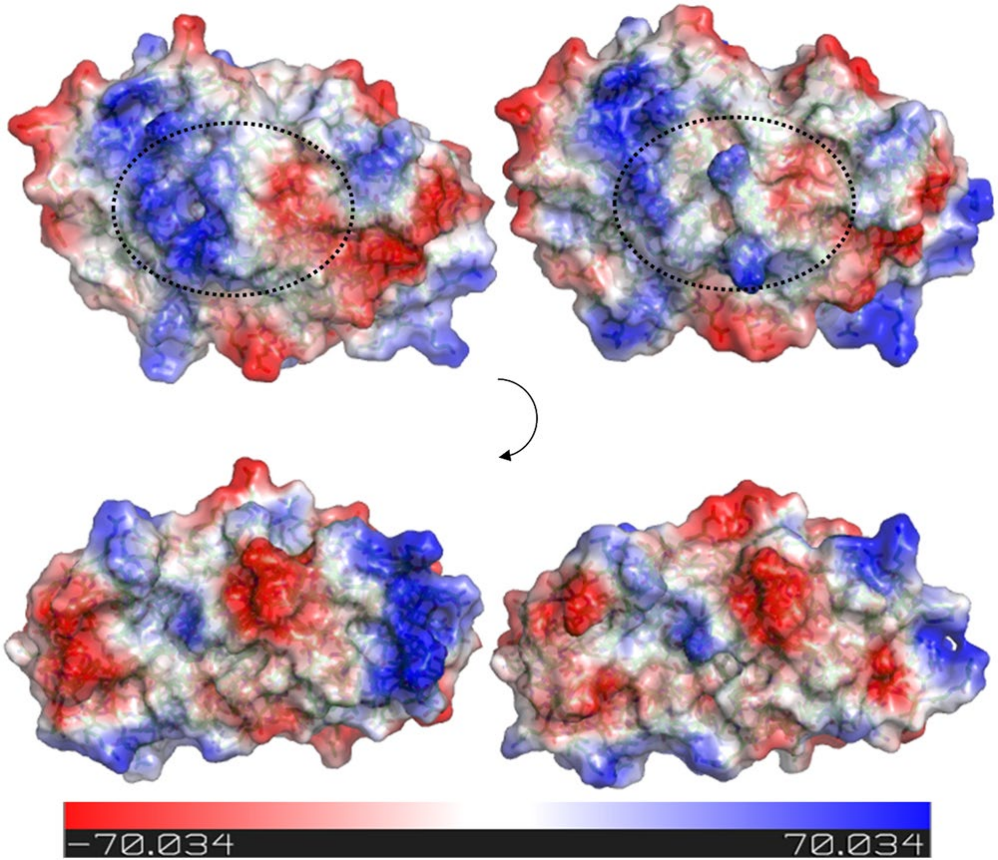
Molecular surface of AioX changes upon arsenite-binding. Molecular surface of AioX, coloured based on the electrostatic potential, is shown for the AioX molecule in absence (left) and in the presence (right) of arsenite. Top view displays the surface changes contributed by the residues comprising 53–61 loop that closes onto the ligand site upon arsenite-binding, with the specific area outlined by dashed circle. Bottom view is of the surface when the molecule on the top was rotated by 90 degrees showing very little effect on that side of the molecule. The two molecules were displayed jointly and the surface electrostatic potentials were calculated simultaneously in order to ensure the consistency in colouring.

Furthermore, conformational rearrangement upon arsenite binding would inevitably result in changes to the molecular surface of AioX, including its electrostatic potential (Fig. 6). Interestingly, while the structures of apo-AioX and the phosphate-bound AioX contained a monomer in the asymmetric unit, the arsenite-bound molecule crystallised with six molecules in the asymmetric unit of the unit cell. Although the six molecules were arranged as the three repeats of a loosely associated dimer none of the interfaces is sufficiently large to be indicative of a biologically significant assembly. It should be noted that while surface residues 59–61 in the arsenite-bound structure are near the neighbouring molecule in the asymmetric unit, they are too distant to be directly involved in the interactions, while in the apo-AioX Asp61 interacts with the side chain of Arg281 from the crystallographic symmetry related molecule. These small differences in molecular packing support the idea that 53–61 loop conformation change might result in the generation of a new protein interaction surface.

### Phosphate-bound and arsenite-bound forms of AioX are not identical

Superposition of the phosphonate-bound structure of PhnD and the phosphate-bound structure of AioX emphasizes the structural similarity between the two proteins, while close inspection of the ligand-binding sites identifies a set of conserved residues that are engaged in interactions with two similar ligands (Figs 4 and 5A). However, the two binding sites are not identical and while one side of the binding pocket contains identical residues, Ser161, Ser163, His192, Tyr88 and Tyr131 specifically (AioX numbering), the other residues in the binding pocket appear to provide substrate specificity. In PhnD, the phosphonate molecule forms an additional hydrogen bond with the side chain of Thr128 (Asn162 in AioX) and a water-mediated interaction with Ser68, while the carboxylate groups of Glu177 and Asp205 interact with the positively charged end of the 2-aminoethyl phosphonate (Tyr212 and Pro240 are present in the corresponding locations in AioX). On the other hand, a phosphate ion found in the binding site of AioX formed AioX-specific hydrogen bonds with Asp210 and Cys106, which in PhnD correspond to Asn175 and Gly65, respectively.

**Figure 7:**
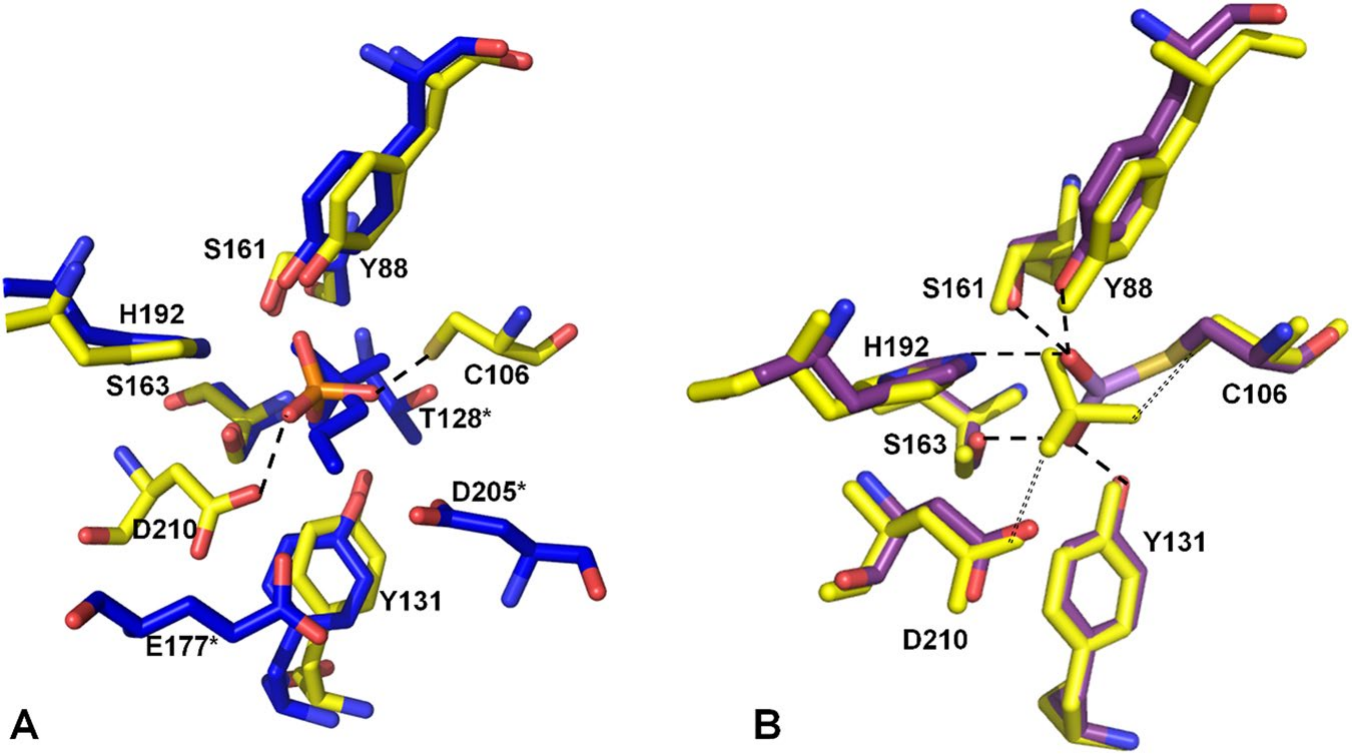
Overlay of the residues forming the ligand binding-sites. (**A**) Comparison of a phosphate-bound AioX (yellow carbon atoms) and the phosphonate-bound PhnD (blue carbon atoms). For clarity, all atoms in the phosphonate molecule are coloured blue. All AioX residues involved in phosphate binding are shown and labelled. The two AioX-specific hydrogen bonds to the phosphate ion are denoted by dashed lines (2.5 Å and 2.6 Å from D210 oxygen and C106 sulphur to phosphate oxygen, respectively). Residues numbered with the asterisk refer to the PhnD structure and they are specific for that protein. (**B**) Comparison of a phosphate-bound AioX (all yellow) and arsenite-bound AioX (purple carbons). Hydrogen bonds with arsenite are denoted by dashed lines while the interactions seen only in the presence of phosphate (same as in 7 A) are shown by dotted lines.

The presence of a phosphate ion in the binding site of AioX leads to the hypothesis that the phosphate ion mimics arsenite binding. A comparison of the X-ray structures however, indicates that while AioX can bind phosphate, the resulting structure does not stabilize the 53–61 loop conformation seen in the arsenite-bound form. Furthermore, even though the structure is similar to the arsenite-bound form, with the main conformation of Tyr88 akin to that of the arsenite-AioX (Fig. 7), electron density for residues 53–61 are ambiguous and due to the disorder, we were unable to confidently build a model for residues 55–57. Within region 55–57 the structure had more similarity to the apo rather than the arsenite-bound form of AioX.

### Conservation of AioX-binding pocket in close homologues

**Figure 8:**
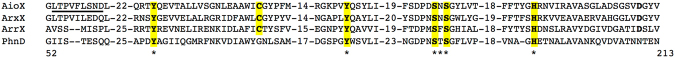
Binding-pocket alignment of AioX, ArrX, ArxX and PhnD. Residues that bind phosphate in AioX are in bold; residues that bind arsenite in AioX are highlighted in yellow; residues that bind phosphonate in PhnD are denoted *; putative signaling loop in AioX is underlined; AioX numbering. Note: there is only one residue that binds phosphate but not arsenite, and one residue that binds phosphonate in PhnD that is not conserved in AioX/ArrX/ArxX.

The residues that constitute the binding pocket of the arsenite- and phosphate-bound structures of AioX, are Tyr88, Cys106, Tyr131, Ser161, Ser163, His192 and Asp210. Alignment of AioX with ArrX, ArxX and PhnD sequences (Fig. 8), shows that residues are conserved in the AioX subfamily of PBPs, indicating similar substrate-binding capabilities. The alignment also highlights the absence of Cys106 in PhnD. The hydrophobic residues which reach towards the core of the protein in arsenite-bound AioX are also present in Arx except Phe57 is an Ile (also hydropobic) in Arx (Fig. 8). However, the corresponding loop regions in ArrX and PhnD are shorter and more polar with AioX-like residues Val56 and Phe57 missing from that part of the sequence (Fig. 8).

### Oxyanion-binding studies demonstrate substrate specificity

**Figure 9:**
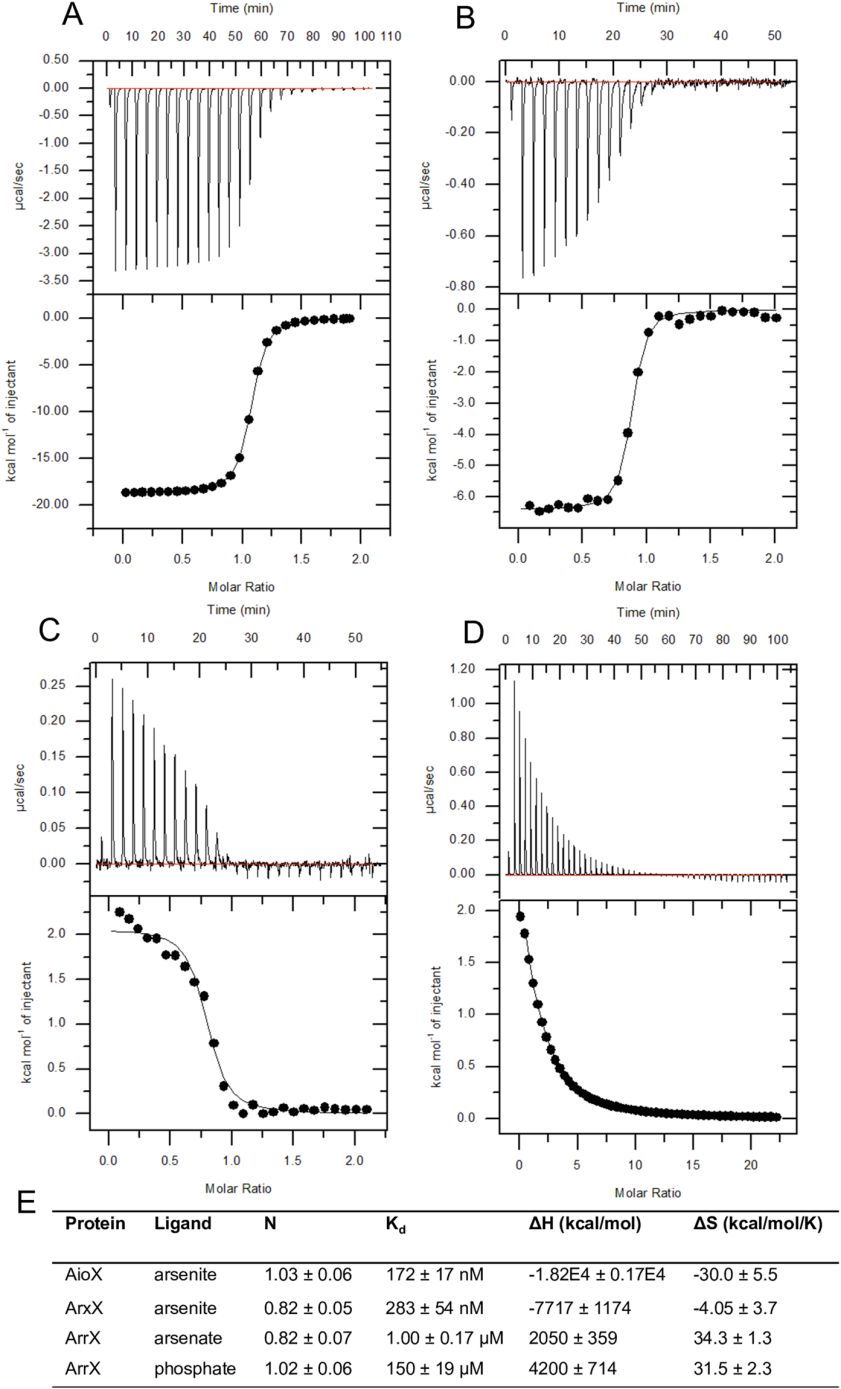
Binding thermodynamics of AioX, ArxX and ArrX with their respective substrates. Upper panels: raw thermograms; lower panels: integrated binding isotherms. (**A**) AioX with arsenite; (**B**) ArxX with arsenite; (**C**) ArrX with arsenate; (**D**) ArrX with phosphate; (**E**) Thermodynamic parameters of substrate binding at 25 °C, values represent mean and standard deviation of three experiments, N: stoichiometry; K_d_: dissociation constant; ΔH: enthalpy change; ΔS: entropy change.

Although the residues involved in binding arsenite and phosphate are conserved in the three orthologues, AioX, ArxX and ArrX (Fig. 8), isothermal titration calorimetry (ITC) experiments indicated distinct substrate specificity of these proteins (Fig. 9). We tested a range of oxyanions including antimonite, arsenate, arsenite, molybdate, phosphate, phosphonate, selenate, sulphate, tetrathionate and thiosulphate as potential ligands for each protein. AioX and ArxX bound arsenite with a K_d_ of 177 and 311 nM, respectively (Fig. 9) and no interaction was detected with any of the other substrates tested (data not shown). It was surprising not to detect phosphate binding to AioX or ArxX given, (1) we solved a structure of AioX with a phosphate ion in the binding pocket (Fig. 4) and (2) that amino acids involved in phosphate binding in the structure of AioX are conserved in ArxX. As we were unable to detect any binding interaction, based on the conditions used in the ITC we estimate that the K_d_ for phosphate would have to be greater than 2 mM (data not shown). ArrX bound arsenate and also phosphate with a K_d_ of 1 and 150 μM, respectively (Fig. 9) while no interaction was detected with any of the other ligands tested (data not shown). Binding of phosphate to ArrX is not surprising given that arsenate and phosphate have analogous chemical structures. Interestingly, ligand-binding affinities for this protein are in micromolar compared to the nanomolar range observed for AioX and ArxX indicating complex tuning of the ion-binding mechanisms, probably involving other structural features beyond direct interactions with the protein side-chains. We used a one-site binding model to fit the data for ArrX with arsenate, as its homology with AioX indicates a single binding pocket, even though the data exhibits a more complex behaviour. A two-site model does fit the data, however the K_d_ values are not replicable between titrations. At pH 8 used in the ITC experiments, the arsenate acquires two forms, H_2_AsO_4_^−^ and HAsO_4_^2−^, which could account for different K_d_ values in the same titration. Alternatively, the arsenate titration thermogram might include a component resulting from the ligand-induced protein-protein interactions. In summary, at physiological concentrations, AioX, ArxX and ArrX bind the substrate that their cognate molybdenum-containing enzyme metabolises.

## Discussion

### Structure and potential signalling mechanism of AioX

AioX is a class II PBP^25^ and a member of the D-cluster of substrate-binding proteins^26^. Proteins of this type predominantly bind tetrahedral oxyanions^26^ and while the binding of arsenate and phosphate by ArrX conforms to this trend, the binding of arsenite by AioX and ArxX is different as arsenite has a trigonal pyramidal geometric structure. The AioX orthologues are the only members of this family that function as signalling proteins. The other members of the family, including the structural homologue PhnD, are part of ABC transporters. Other PBPs shown to be involved in signal transduction include LuxP and TorT, which bind autoinducer-2^29^ and trimethylamine^27^ and regulate the *lux*^30^ and *tor*^28^ operons, respectively. LuxP and TorT however are structurally and phylogenetically distinct to AioX, indicating independent evolutionary origins.

The arsenite-bound crystal structure of AioX showed significant conformational changes compared with the apo and phosphate-bound structures, specifically in the loop of residues 53–61 and the shape, as well as electrostatic potential, of the protein surface. It is possible that these modifications allow its interaction with the sensor histidine kinase, AioS, leading to its activation. The fact that the arsenite-bound and apo-AioX crystals displayed differences in molecular packing (forming weak local and crystallographic symmetry related dimers, respectively) supports the idea that loop conformation change might result in the generation of a new protein interaction surface.

### Substrate specificity

Binding experiments performed by ITC showed that AioX and ArxX could only bind arsenite from the potential ligands selected, while Arr bound arsenate and phosphate (although the latter with a 150 times higher K_d_). Phosphate concentrations that organisms encounter will depend on the environment. *A. ehrlichii* MLHE-1 is from an environment extremely rich in phosphate (up to 1 mM)^31^, while gold mine tailings often don’t contain enough phosphate to support microbial growth^32^, and eutrophied lake sediments may contain 7 µM phosphate^33^ (both *Rhizobium* sp. str. NT-26 and *C. arsenatis* were isolated from mining environments). A phosphate ion was identified in the crystal structure of AioX, however, its binding does not stabilize the putative signalling loop as is the case with arsenite binding. As AioX and ArxX did not bind phosphate in conditions tested in ITC, we conclude that AioX and ArxX do not bind phosphate under biologically relevant conditions.

The binding residues involved in arsenite binding in AioX (and presumably ArxX) are also conserved in ArrX, which did not bind arsenite but did bind arsenate and phosphate albeit the latter with about 150-fold less affinity. This finding indicates that binding residues alone do not confer ligand specificity but other factors, such as overall conformation of the protein, may play a role in selectivity. The K_d_ of ArrX for phosphate indicates that in some high phosphate environments expression of the arsenate reductase genes may be inhibited.

### Evolution of the *XSR* regulatory cluster

Based on phylogenetic studies, some molybdenum-containing enzymes were present in the last universal common ancestor^34^ and it is hypothesised that Aio is the common ancestor to all other members of the DMSO reductase family of molybdoenzymes^34,35^. Studies have also shown that while respiratory molybdoenzymes are principally inherited by vertical gene transfer, they are also subject to duplication events and divergence, particularly in the *Firmicutes* and *Proteobacteria* phyla^36^. Such a scenario is apparent for divergence of Arx and Arr, where the ‘older’ enzyme Arr, which uses menaquinone to reduce arsenate, has diverged to form Arx which uses ubiquinone to oxidise arsenite^37^.

The congruency of the AioX, AioS and AioR phylogenies to the 16S rRNA gene indicates that the regulatory genes emerged early in prokaryotic evolution and their functions adapted to that of the molybdoenzymes, whose gene expression they regulate. This is supported by the similar GC content of the regulatory genes and the molybdoenzyme genes (data not shown). Adaptation to conform to the function of a cognate molybdoenzyme is also observed in the NarJ family of proteins, which function as chaperones and are essential for cofactor insertion in several families of molybdoenzymes^38^.

The *aioXSR* genes are present in some arsenite oxidisers of the *Alpha-* and *Betaproteobacteria*. In *Polaromonas* sp. str. GM1, the Aio is constitutively expressed and a putative phage integrase is present immediately upstream of *aioB* suggesting that this insertion event may have led to the deletion of the *aioXSR* genes^39^. We have identified other examples of gene loss including in the putative arsenite oxidiser *Verminephrobacter aporrectodeae*^40^ where a 188 bp fragment of the *aioX* gene remains, with the rest of the regulatory cluster missing. The putative arsenite oxidisers of the genus *Burkholderia* (member of the *Betaproteobacteria*) also show how the *aioXSR* genes may have been lost. The *aioXSR* genes are present in *B. cenocepacia* but absent in *B. vietnamensis, B. multivorans* and *B. ubonensis*. Typically, AioA and AioB phylogenies resemble those of the 16S rRNA gene consistent with vertical inheritance^34,35^ which is the case for *B. cenocepacia* but not the other *Burkholderia* species. In the other *Burkholderia* species, the AioA subunit clusters with six *Pseudomonas* (*Gammaproteobacteria*, which lack *aioXSR*) species. Transposases and integrases are located within 10 kb of the *aioBA* genes in these *Burkholderia* species, which may indicate loss and horizontal gene transfer of the *aioBA* genes from *Pseudomonas* to *Burkholderia* accounting for the absence of *aioXSR* in those species.

The *XSR* gene cluster is conserved in all *arx*-containing organisms but it appears that it has been lost in some that contain *aio*. Phylogenetic analyses of the *arrAB* genes suggests both vertical and horizontal transfer of these genes^35^ and it’s possible that the regulatory genes were lost after such a horizontal gene transfer event.

In conclusion, we propose that the protein AioX represents a new subfamily of PBPs that bind arsenic oxyanions.

## Methods

### Cloning of *aioX* and its orthologues

The *aioX* gene from *Rhizobium* sp. str. NT-26 and its orthologues, *arxX* from *A. ehrlichii* str. MLHE-1 and *arrX* from *C. arsenatis* were cloned without their signal sequences into pPROEX-HTb+ (Invitrogen) for cytoplasmic protein expression in *E. coli* str. JM109. The *aioX* and *arrX* genes were amplified from genomic DNA using PCR with the following primers: *aioX*F, *aioX*R, *arrX*F and *arrX*R (Supplementary Table S1) whereas a codon-optimised version of the *arxX* gene was synthesised (Supplementary Fig. S1, GeneArt, ThermoFisher Scientific) and then cloned into pPROEX-HTb+. Expression constructs were confirmed by sequencing (GATC Biotech).

### Heterologous expression and purification of AioX and its orthologues

AioX, ArxX and ArrX were heterologously expressed in 1 l lysogeny broth (LB) (in a 5 l flask) containing 100 µg/ml ampicillin and 40 µM isopropyl β-D-1-thiogalactopyranoside (IPTG) at 21 °C with shaking at 180 rpm for 20–24 h. Recombinant proteins were purified from JM109λ*pir* using affinity and size-exclusion chromatography. Cells were harvested by centrifugation at 5000 × *g* for 10 min (4 °C) and the pellets suspended in 40 ml binding buffer (500 mM NaCl, 20 mM potassium phosphate, 20 mM imidazole, pH 7.3). Cells were centrifuged at 16,000 × *g* for 10 min (4 °C), suspended in 10 ml/gm (wet weight) binding buffer and broken at 14,000 PSI in a pressure cell homogenizer (Stansted Fluid Dynamics). Debris and unbroken cells were removed by centrifugation at 30,000 × *g* for 30 min (4 °C). Total cell extract was applied to a 1 ml His Gravitrap (GE Healthcare) Ni-charged column which was then washed with 100 ml binding buffer. Bound proteins were eluted from the column in 5 ml elution buffer (500 mM NaCl, 20 mM potassium phosphate, 200–500 mM imidazole, pH 7.3). The eluate was filtered through a 0.22 µm filter (Millipore), concentrated using a 10,000 MWCO centrifugal filter (Millipore) and loaded onto a Superdex 75 gel filtration column (GE healthcare) pre-equilibrated with either 20 mM Tricine, pH 7.5 (AioX and ArxX) or 50 mM Tris-HCl, pH 8 (ArrX). Chromatography was carried out at 0.8 ml/min and fractions corresponding to the protein peaks were pooled and concentrated with the 10,000 MWCO centrifugal filter (Millipore), snap frozen in liquid nitrogen and stored at −80 °C until required. Protein concentrations were determined with the absorbance at 280 nm, measured with a NanoDrop 2000 spectrophotometer (Thermo), and a predicted molar absorbance coefficient (ExPASy, Swiss Institute of Biofinformatics)^41,42^; ε (M−1 cm^−1^) for AioX, 42860, ArxX 44350, ArrX 25330. Protein purity was confirmed by sodium dodecyl sulphate-polyacrylamide gel electrophoresis.

For crystallisation, the His-tag was removed from AioX using AcTEV protease (Invitrogen) according to the manufacturer’s instruction. The cleaved AioX was then separated from uncleaved protein and the protease using affinity chromatography as described above.

### Selenomethionine labelling of the recombinant NT-26 AioX

The sulphur atoms of methionine residues in AioX were replaced with selenium by providing selenomethionine in the growth medium during protein expression using a modified protocol^43^. JM109 harbouring pPROEX-HTb + -*aioX* was grown at 37 °C overnight in 50 ml of M9 medium^44^ containing ampicillin (100 μg/ml). The 50 ml was inoculated into 1 l M9 medium and incubated at 37 °C with shaking (170 rpm). Cells were grown until OD_600 nm_ ~0.5 followed by the addition of an amino acid mix consisting of 100 mg each of lysine, threonine, phenylalanine; 50 mg each of leucine, isoleucine, valine and 60 mg of L-selenomethionine and the cells grown for 15 minutes prior to induction with IPTG. The SeMet-AioX protein was purified as described above with one modification, all buffers contained 5 mM of dithiothreitol.

### Crystallisation of NT-26 AioX, data collection and structure determination

For crystallisation of the SeMet-AioX, apo-AioX and AioX-arsenite, the recombinant AioX protein was concentrated to 20 mg/ml. Crystallization screening was performed at 16 °C, using a mosquito (TTP Labtech) and the crystallisation screens from Hampton Research (Index) and Molecular Dimensions (Structure 1&2, PACT and JCSG). Conditions that yielded crystals were as follows: (1) SeMet-AioX crystals grew in 0.49 M NaH_2_PO_4_ and 0.91 M K_2_HPO_4_ pH 7.2, (2) apo-AioX crystals grew in 20% PEG 3350, 0.2 M sodium malonate and 0.1 M Bis-Tris propane, pH 8.5 and, (3) AioX-arsenite crystals grew in 25% PEG 3350 and 0.1 M Tris-HCl, pH 7.6. Single crystals were collected and mounted in a nylon cryo-loop using 25% glycerol as a cryo-protectant and flash cooled directly in liquid nitrogen prior to data collection.

Crystals were screened and X-ray diffraction data collected at the following synchrotron radiation sources: Diamond Light Source, beamlines I04 and I02 and Soleil synchrotron, beamline Proxima 1. The diffraction images were integrated and scaled using the XDS software package and merged using Aimless in the CCP4 program suite^45^. SAD data were collected from one SeMet-AioX crystal and processed with CCP4 software. The MTZ reflection file obtained was used to determine the position of five selenium atoms through SHELX C/D/E (CCP4). BUCCANEER was used to generate the AioX model. The final AioX model was generated through interactive cycles of model building in Coot^46^ and a restrained refinement routine in REFMAC5^47^. The structures of apo-AioX and arsenite-bound AioX were solved using molecular replacement using the phosphate-bound structure as the model, but with the phosphate ion omitted. Phases were obtained with PhaserMR^48^ and model building was performed in Coot^46^. Refinement of the new structure was carried out in REFMAC5^47^. The data collection, processing and refinement statistics are listed in Table 1. All figures of crystal structures and molecular surface calculations were prepared using PyMOL^49^. The following PDB entries were used for structural comparisons: 3N5L (a binding protein component of an ABC phosphonate transporter from *Pseudomonas aeruginosa*) and 3P7I (a PhnD in complex with 2-aminoethyl phosphonate from *E. coli*)^50^. Three structures and the associated structure factors were submitted to PDB with the following IDs: 6ESK, 6EU7 and 6ESV for apo, arsenite-bound and a phosphate-bound AioX.

### Substrate-binding thermodynamics determined by isothermal titration calorimetry

Binding of potential substrates by AioX, ArxX and ArrX was determined by ITC using a MicroCal200 ITC instrument (Malvern) at 25 °C with constant stirring of 750 rpm. Binding parameters were calculated using Microcal Origin software with a one-site binding model to give estimated values of N, K_*d*_, ΔH, and ΔS. Antimonite (antimonyl tatrate; C_8_H_10_K_2_O_15_Sb_2_), arsenate (Na_2_HAsO4), arsenite (NaAsO_2_), molybdate (Na_2_MoO_4_), phosphate (KH_2_PO_4_), phosphonate (2-aminoethyl phosphonic acid; PO_3_H_2_(CH_2_)_2_NH_2_), selenate (Na_2_SeO_4_), sulphate (Na_2_SO_4_), tetrathionate (K_2_S_4_O_6_)_,_ thiosulphate (Na_2_S_2_0_3_) were tested as potential substrates by titrating 1–10 mM substrate into 100 µM protein in 1.5–2 µl injections. Protein-substrate combinations that tested positive for binding were repeated with three independent protein preparations. AioX and ArxX titrations were performed in 20 mM Tricine, pH 7.5 and ArrX in 50 mM Tris-HCl, pH 8. Substrates were prepared in the same buffer as the protein into which they were titrated.

### Phylogenetic analysis

Sequences from confirmed arsenic metabolisers and other representatives were retrieved from GenBank using BLAST^51^ with NT-26 sequences as queries. Phylogenetic analyses were performed using MEGA^52^. Multiple sequence alignments were generated using MUSCLE^53^. Phylogenetic trees were constructed with the maximum-likelihood method, using the Jones-Taylor-Thornton model, and tested by 200 bootstrap resamples. Accession numbers used to construct trees are in Supplementary Table S2.

### Data availability statement

All data generated or analysed during this study are included in this published article (and its Supplementary Information file).

## Electronic supplementary material

Supplementary information
