# Supplementary material for: A new family of periplasmic-binding proteins that sense arsenic oxyanions
Source: Sci Rep. 2018 Apr 19;8:6282. doi: 10.1038/s41598-018-24591-w (PMC5908839; doi:10.1038/s41598-018-24591-w)
Supplement: Supplementary file 1 — Supplementary information [file 41598_2018_24591_MOESM1_ESM.docx]

**A new family of periplasmic-binding proteins that sense arsenic oxyanions**

Consuelo Badilla, Thomas H. Osborne, Ambrose Cole, Cameron Watson, Snezana Djordjevic, Joanne M. Santini

**Table S1.** Primers used for cloning *aioX* and its orthologues

| **Primer** | **Sequence** |
| --- | --- |
| AioXF | 5'-GCGGATCCACTGTCGGGCTTACCGCATTG-3' |
| AioXR | 5'-GCGAATTCCTCATCCCAGCCTCCGCACGCG-3' |
| ArrxF | 5'-GCGGATCCTCGGTAAAACCTATTCCGGTT-3' |
| ArrXR | 5'-GCAAGCTTCTACTCAACCACCTCTATTTT-3' |

Restriction sites are underlined. *Bam*HI was used in conjunction with *Eco*RI and *Hin*dIII to clone *aioX* and *arrX,* respectively.

**Figure S1.** Nucleotide sequence of codon-adapted *arxX*

GCAGAACATGCAGCAGAACAGAGCCAGCCGCTGCGTATTGGTCTGACACCGGTTATTCTGGAAGATCAGCTGAGCTTTCTGGATGAATGGCAGACCTATCTGGAACGTCGTACCGGTCGTGAAGTTCAGTTTGTTCGTCGTAATAGCTATGGTGAAGTTGTTGAACTGGCACTGCGTGGTCGTATTGATTTTGCATGGCTGTGTGGTTATCCGTATGTTCAGCATGATACCCGTCTGGGTCTGCTGGCAGTTCCGCGTTTTAATGGTGCACCGCTGTATCAGGCATATCTGATTGTTCCGGCAAATGATCAGGCAACCGAAAGCATTAAAGATCTGGCAGGTCGTGTTTTTGCATATAGCGATCCGAATAGCAATAGCGGTTGGCTGTATGGTCAGCAACTGCTGGATGAAGCCGGTGTTATGGACCCGGAACGTTTTTTTCTGCGTACCTTTTTTACCTTTAGCCACCGTAAAGTTGTGGAAGCAGTTGCAGAACGTGTTGCACATGGTGGTCTGGTTGATGGTTATGTTTGGGAAACCCTGGCACTGGATCGTCCGGATCTGACCGGTCGTACCCGTGTTGTTCATCGTAGCGAACCGTTTGGTTTTCCGCCTCTGGTTGCAAGTCCGGGTACAACCGAAGGTGAACGTCGTGCACTGCAAGAGGCACTGTTTGGTATGGATGAGGATCAGCGTGGTCGCGAGCTGCTGGAACGCCTGAACCTGGAACGCTTTGAAGCGGGTGATGAAAGCCTGTATGATGGTATTCGTGATCTGAAACGTCTGGTGGATGTTGGTCGTCTGCCGGAACCTCCGGATGGCTATGAACGT

**Table S2.** Accession numbers and identifiers for sequences used to construct phylogenetic trees.

| **Organism** | **16S rRNA gene** | **Mo** | **AioX** | **AioS** | **AioR** |
| --- | --- | --- | --- | --- | --- |
| **Aio** |  |  |  |  |  |
| *Achromobacter* *arsenitoxydans* str. SY8 | EU073119.1 | ABP63660.1 | ACJ83257.1 | ACJ83256.1 | ACJ83255.1 |
| *Agrobacterium tumefaciens* str. 5A | AF388033.1 | EHJ95436.1 | EHJ95440.1 | EHJ95439.1 | EHJ95438.1 |
| *Alcaligenes faecalis* | QWA_r17303 | AAQ19838.1 | AAQ19840.1 | AAQ19841.1 | AAQ19842.1 |
| *Bosea* sp. str. WAO | DQ986321.1 | KUL93390.1 | KUL93386.1 | KUL93387.1 | KUL93388.1 |
| *Herminiimonas arsenicoxydans* str. ULPAs1 | NR_125502.1 | CAL60690.1 | CAL60692.1 | CAL60693.2 | CAL60694.1 |
| *Rhizobium* sp. str. NT-26 | AF159453.1 | WP_052642472.1 | CCF22051.2 | WP_052642465.1 | WP_052642467.1 |
| *Sinorhizobium* sp. str. M14 | EF442029.1 | YP_007974270.1 | WP_015647696.1 | YP_007974273.1 | YP_007974272.1 |
| *Starkeya novella* | NR_074219.1 | WP_013166106.1 | ADH88605.1 | WP_013166109.1 | WP_013166108.1 |
| **Arr** |  |  |  |  |  |
| *Bacillus selenatarsenatis* | NR_041465.1 | WP_041964264.1 | GAM12299.1 | WP_052442054.1 | WP_041964259.1 |
| *Bacillus selenitireducens* | NR_028707.1 | WP_013173528.1 | WP_013173529.1 | WP_013173530.1 | WP_013173531.1 |
| *Chrysiogenes arsenatis* | NR_029283.1 | WP_027389593.1 | WP_051321412.1 | WP_051321411.1 | WP_051321410.1 |
| *Desulfitobacterium dichloroeliminans* | AJ565938.1 | WP_015261495.1 | AGA68500.1 | WP_015261498.1 | WP_015261497.1 |
| *Desulfitobacterium hafniense* | AB049340.1 | BAE85920.1 | BAE85916.1 | BAE85917.1 | BAE85918.1 |
| *Desilfosporosinus youngiae* | DQ117470.1 | WP_007781366.1 | EHQ88674.1 | WP_007781362.1 | WP_007781363.1 |
| *Natranaerobius thermophilus* | NR_074181.1 | WP_012447122.1 | ACB84245.1 | WP_012447128.1 | WP_012447127.1 |
| *Sulfurospirillum arsenophilum* | NR_044806.1 | WP 041956283.1 | WP_041956280.1 | WP_041956277.1 | WP_041956274.1 |
| *Sulfurospirillum barnesii* | NR_028692.1 | WP_014770549.1 | WP_014770547.1 | WP_014770546.1 | WP_014770545.1 |
| *Sulfurospirillum multivorans* | NR_121740.1 | WP_025346199.1 | AHJ14370.1 | WP_025346196.1 | WP_025346195.1 |
| **Arx** |  |  |  |  |  |
| *Alkalilimnicola ehrlichii* str. MLHE-1 | NR_074775.1 | ABI55571.1 | ABI55573.1 | ABI55574.1 | ABI55575.1 |
| *Ectothiorhodospira* sp. str. BSL-9 | ECTOBSL9_RS08185 | ANB03234.1 | ANB03920.1 | ANB03232.1 | ANB03231.1 |
| *Ectothiorhodospira* sp. str. PHS-1 | EU590916.1 | EHQ52454.1 | EHQ52452.1 | EHQ52451.1 | EHQ52450.1 |
| *Magnetospirillum magnetotacticum* | AB680822.1 | WP_041039509.1 | WP_041039533.1 | WP_009868505.1 | WP_052472884.1 |
| *Sulfuricella denitrificans* | AB506456.1 | WP_009207744.1 | WP_009207746.1 | WP_041674374.1 | WP_009207748.1 |
| *Thioalkalivibrio nitratireducens* | NR_102486.1 | AGA32636.1 | AGA32639.1 | AGA32640.1 | AGA32641.1 |
| *Thioalkalivibrio sulphidiphilus* | GU735094.1 | WP_026289649.1 | WP_018952914.1 | WP_026289648.1 | WP_018952912.1 |
| *Thiocapsa* sp. str. KS-1 | EF581005.1 | CRI67721.1 | CRI67718.1 | CRI67717.1 | CRI67716.1 |
| **Dms** |  |  |  |  |  |
| *Halomonas halodenitrificans* | - | WP_043488660.1 | - | - | - |
| *Rhodovolum sulfidophilum* | - | Q8GPG4.1 | - | - | - |
| *Sagittula stellata* | - | WP_040604859.1 | - | - | - |
| **Dor** |  |  |  |  |  |
| *Escherichia coli* | - | KIG54607.1 | - | - | - |
| *Salmonella enterica* | - | KNB34701.1 | - | - | - |
| **Ebd** |  |  |  |  |  |
| *Aromatoleum aromaticum* | - | 2IVF_A | - | - | - |
| **Fdh** |  |  |  |  |  |
| *Cupriavidus necator* | - | KUE90306.1 | - | - | - |
| *Escherichia coli* | - | 1KQG | - | - | - |
| *Pseudomonas aeruginosa* | - | YP_003933614.1 | - | - | - |
| *Shewanella oneidensis* | - | NP_720033.1 | - | - | - |
| **Nap** |  |  |  |  |  |
| *Escherichia coli* | - | NP_416710.1 | - | - | - |
| *Pseudomonas aeruginosa* | - | NP_249865.1 | - | - | - |
| *Rhodobacter spaeroides* | - | AAC23522.1 | - | - | - |
| **Nar** |  |  |  |  |  |
| *Escherichia coli* | - | EGT67762.1 | - | - | - |
| *Pseudomonas aeruginosa* | - | NP_252564.1 | - | - | - |
| *Streptomyces coelicolor* | - | NP 629099.1 | - | - | - |
| **Nas** |  |  |  |  |  |
| *Pseudomonas aeruginosa* | - | NP_250470.1 | - | - | - |
| *Synechocystis* sp. str. PCC6803 | - | WP_010872118.1 | - | - | - |
| **Pcr** |  |  |  |  |  |
| *Azospira suillum* | - | 4YDD_A | - | - | - |
| *Dechloromonas aromatica* | - | WP_011288314.1 | - | - | - |
| *Sedimenticola selenatireducens* | - | WP_037375984.1 | - | - | - |
| **Phs** |  |  |  |  |  |
| *Desulfovibrio vulgaris* | - | WP_010937484.1 | - | - | - |
| *Shewanella oneidensis* | - | NP_719592.1 | - | - | - |
| **Psr** |  |  |  |  |  |
| *Thermus thermophilus* | - | 2VPZ_A | - | - | - |
| *Wolinella succiogenes* | - | WP_011138081.1 | - | - | - |
| **Ser** |  |  |  |  |  |
| *Sedimenticola selenatireducens* | - | WP_029134803.1 | - | - | - |
| *Thauera selenatis* | - | Q9S1H0.1 | - | - | - |
| **Tor** |  |  |  |  |  |
| *Escherichia coli* | - | NP_416386.4 | - | - | - |
| *Rhodobacter capsulatus* | - | 3DMR | - | - | - |
| *Shewanella oneidensis* | - | NP_716855.1 | - | - | - |
| **Ttr** |  |  |  |  |  |
| *Salmonella enterica* | - | CAB37416.1 | - | - | - |
| **ABP** |  |  |  |  |  |
| *Mycobacterium tuberculosis* | - | - | 1PC3 | - | - |
| **MBP** |  |  |  |  |  |
| *Archaeoglobus fulgidis* | - | - | 2ONR | - | - |
| *Azotobacter vinelandii* | - | - | 1ATG | - | - |
| *Escherichia coli* | - | - | 1WOD | - | - |
| *Methanocaldococcus jannaschii* | - | - | 3CFZ | - | - |
| *Methanosarcina acetivorans* | - | - | 3CFX | - | - |
| *Pyrococcus furiosus* | - | - | 3CG1 |  |  |
| *Pyrococcus horikoshii* | - |  | 3CG3 | - | - |
| *Xanthomonas citri* | - | - | 2H5Y | - | - |
| **PBP** |  |  |  |  |  |
| *Chlostridium perfringens* | - | - | 4GD5 | - | - |
| *Escherichia coli* | - | - | 1IXH | - | - |
| *Lactobacillus brevis* | - | - | 4ECF | - | - |
| *Mycobacterium tuberculosis* | - | - | 4LVQ | - | - |
| *Pseudomonas aeruginosa* | - | - | 4OMB | - | - |
| *Pseudomonas fluorescens* | - | - | 4F18 | - | - |
| *Strenotrophomonas*  *maltophilia* | - | - | 5JK4 | - | - |
| *Streptococcus pneumoniae* | - | - | 4H1X | - | - |
| *Vibrio cholerae* | - | - | 1TWY | - | - |
| *Xanthomonas citri* | - | - | 5I84 | - | - |
| *Yersinia pestis* | - | - | 2Z22 | - | - |
| **PhnD** | - | - |  | - | - |
| *Escherichia coli* | - | - | 3QUJ | - | - |
| *Pseudomonas aeruginosa* | - | - | 3N5L | - | - |
| **SBP** | - | - |  |  |  |
| *Salmonella enterica* | - | - | 1SBP | - | - |
| *Xanthomonas citri* | - | - | 5UM2 | - | - |
| **TBP** | - | - |  |  |  |
| *Geobacter sulfurreducens* | - | - | 3LR1 | - | - |

Mo: molybdenum-containing subunit of DMSO reductase family of molybdoenzymes; AioX: periplasmic/substrate-binding protein; AioS: sensor histidine kinase; AioR: response regulator; ABP: antigen binding protein; Aio and Arx: aerobic and anaerobic arsenite oxidase, respectively; Arr: arsenate respiratory reductase; Dms: dimethylsulphide dehydrogenase; Dor: DMSO reductase; Ebr: ethylbenzene dehydrogenase; Fdh: formate dehydrogenase; MBP: molybdate-binding proteins; Nap: periplasmic nitrate reductase; Nar; respiratory nitrate reductase; Nas: assimilatory nitrate reductase; PBP: phosphate-binding proteins; Pcr: perchlorate reductase; PhnD: phosphonate-binding proteins; Phs: thiosulphate reductase; Psr: polysulphide reductase; SBP: sulphate-binding proteins; Ser: selenate reductase; TBP: tungstate binding protein; Tor: TMAO reductase; Ttr: tetrathionate reductase.

**Figure S2.** Maximum-likelihood phylogenetic tree of 16S rRNA genes from organisms with AioXSR homologues. Red: AioXSR; Blue: ArxXSR; Green: ArrXSR. α: *Alphaproteobacteria*; β: *Betaproteobacteria*; γ: *Gammaproteobacteria*; ε: *Epsilonproteobacteria*; C: *Chrysiogenetes*; F: *Firmicutes*. . Scale bar represents 0.05 substitutions per position. Significant bootstrap values from 200 resamples are shown. Tree was rooted with sequence from *Sulfolobus tokodaii*.
